# Sepsis related mortality of extremely low gestational age newborns after the introduction of colonization screening for multi-drug resistant organisms

**DOI:** 10.1186/s13756-020-00804-8

**Published:** 2020-08-26

**Authors:** Christoph Härtel, Kirstin Faust, Ingmar Fortmann, Alexander Humberg, Julia Pagel, Clara Haug, Reinhard Kühl, Bettina Bohnhorst, Sabine Pirr, Dorothee Viemann, Arne Simon, Michael Zemlin, Silvia Poralla, Andreas Müller, Natascha Köstlin-Gille, Christian Gille, Matthias Heckmann, Jan Rupp, Egbert Herting, Wolfgang Göpel

**Affiliations:** 1 Department of Pediatrics, University of Lübeck, Lübeck, Germany; 2 German Center for Infection Research (DZIF), Partner Site Hamburg-Lübeck-Borstel-Riems, Lübeck, Germany; 3 German Neonatal Network (GNN), Lübeck, Germany; 4 Priming Immunity at the beginning of life (PRIMAL) Consortium, Lübeck, Germany; 5 Department of Pediatrics, University of Würzburg, Josef-Schneider-Strasse 2, D-97080 Würzburg, Germany; 6 Department of Pediatrics, Diakonissenkrankenhaus, Flensburg, Germany; 7 Department of Neonatology, Hannover Medical School, Hannover, Germany; 8 Department of Pediatrics, Saar University Homburg, Homburg, Germany; 9 Department of Pediatrics, University of Bonn, Bonn, Germany; 10 Department of Neonatology, Tuebingen University Children’s Hospital, Tuebingen, Germany; 11 Department of Neonatology and Pediatric Intensive Care, University of Greifswald, Greifswald, Germany; 12 Department of Infectious Diseases and Medical Microbiology, University of Lübeck, Lübeck, Germany

**Keywords:** Colonization screening, Multi-drug resistant organisms, Extremely preterm infants, Sepsis, Sepsis mortality, Preterm infant

## Abstract

**Background:**

In 2013 German infection surveillance guidelines recommended weekly colonization screening for multidrug-resistant (MDRO) or highly epidemic organisms for neonatal intensive care units (NICUs) and extended hygiene measures based on screening results. It remains a matter of debate whether screening is worth the effort. We therefore aimed to evaluate sepsis related outcomes before and after the guideline update.

**Methods:**

The German Neonatal Network (GNN) is a prospective cohort study including data from extremely preterm infants between 22 + 0 and 28 + 6 gestational weeks born in 62 German level III NICUs.

**Results:**

Infants treated after guideline update (*n* = 8.903) had a lower mortality (12.5% vs. 13.8%, *p* = 0.036), reduced rates for clinical sepsis (31.4 vs. 42.8%, *p* <  0.001) and culture-proven sepsis (14.4% vs. 16.5%, *p* = 0.003) as compared to infants treated before update (*n* = 3.920). In a multivariate logistic regression analysis, nine pathogens of culture-proven sepsis were associated with sepsis-related death, e.g. *Pseudomonas aeruginosa* [OR 59 (19–180), *p* <  0.001)]. However, the guideline update had no significant effect on pathogen-specific case fatality, total sepsis-related mortality and culture-proven sepsis rates with MDRO. While the exposure of GNN infants to cefotaxime declined over time (31.1 vs. 40.1%, *p* <  0.001), the treatment rate with meropenem was increased (31.6 vs. 26.3%, *p* <  0.001).

**Conclusions:**

The introduction of weekly screening and extended hygiene measures is associated with reduced sepsis rates, but has no effects on sepsis-related mortality and sepsis with screening-relevant pathogens. The high exposure rate to meropenem should be a target of antibiotic stewardship programs.

## Background

Neonatal sepsis has a major impact on mortality and long-term morbidity. There is an urgent need for new strategies to prevent sepsis, particularly in preterm infants who are highly vulnerable to be involved in healthcare-associated infection outbreaks [1–7]. Endogenous risk factors of sepsis in preterm infants include immaturity of barriers (skin, mucosa) and systemic immune responses while the colonizing microbiota is also considered to play a key role. As a consequence of several infection outbreaks in German NICUs being a topic in the public media [8–10] the German Commission on Hospital Hygiene and Infection Prevention (KRINKO) at the Robert-Koch Institute, Berlin, decided to update the recommendations for infection surveillance in neonatal intensive care units (NICUs) [11]. The 2013 update recommended weekly colonization screening followed by proactive hygiene interventions based on screening results to be mandatory for German NICUs. This strategy aimed to (a) adjust empirical anti-infective therapy in colonized patients when sepsis is suspected, (b) to assess local epidemiology of multi-drug resistant organisms (MDRO) and to recognize an outbreak as early as possible and (c) to prevent spreading of MDRO or pathogens with high epidemic potential by extended hygiene measures [12]. The scientific evidence of this recommendation is limited. Whether colonization is related to sepsis risk with the same pathogen is still a matter of controversial discussion [13–15]. While other European countries have not officially adopted the colonization screening strategy in guidelines of neonatal societies, several units have voluntarily implemented this approach for infection control [16–19]. The German Neonatal Network (GNN) is a population-based, prospective study including 62 tertiary (highest) care level NICUs, i.e. data from > 30% of extremely preterm infants born in Germany. The updated KRINKO recommendations were implemented in all GNN sites at the same time. We aimed to evaluate the sepsis incidence and pathogen specific fatality rates before and after the guideline update.

## Methods

### Cohort of preterm infants

The German Neonatal Network (GNN) is a population-based cohort study of preterm infants < 29 weeks of gestation or < 1000 g birth weight born in 62 neonatal intensive care units in Germany. For this observational study we included all preterm infants born in GNN centers between 1st January 2011 and 31st December 2018 with a gestational age between 22 + 0 and 28 + 6 weeks who were discharged or died before 1st January 2019. Infants with lethal malformations were excluded. For those infants born in GNN centers but not enrolled in GNN (causes for non-enrollment: parents not asked for participation, parents’ refusal or early death of infant) a basic data set including birth weight, gestational age, and major outcomes (blood-culture proven sepsis including causative pathogen and death/cause of death) was collected. For those infants who were enrolled in the GNN by the attending physicians a predefined clinical data set of 250 parameters (including exposure to specific antibiotics) was recorded on case report forms and sent to the GNN coordinating center in Lübeck. Data were managed including double entry of data, providing queries on missing or inaccurate data and plausibility check. For final data validation, a physician specialized in neonatology monitored the data quality by annual on-site visits. Missing data were not imputed.

### Ethics

The study was approved by the ethics committee of the University of Lübeck (08–022) and the local ethical committees at each study center. Written informed consent was obtained from at least one parent on behalf of the infant enrolled in our study.

### Definitions

*Stratification of infants:* The infants were stratified to NICU treatment (survival to hospital discharge or death during initial stay in hospital) before (2011–2013) and after (2014–2018) the guideline update.

*Gestational age* was calculated from the best obstetric estimate based on early prenatal ultrasound and obstetric examination. *Small for gestational age (SGA)* was defined as birth weight percentile < 10 according to gestational age.

*Neonatal sepsis* was defined according to the criteria of the national infection surveillance system “NEO-KISS” [20]. *Clinical sepsis* was defined as sepsis with at least two clinical signs (temperature > 38 °C or < 36.5 °C, tachycardia > 200/min, new onset or increased frequency of bradycardias or apneas, hyperglycemia > 140 mg/dl, base excess <− 10 mval/l, pale/grey skin color, increased oxygen requirements) or one clinical and one laboratory sign (C-reactive protein > 1 mg/dl, immature neutrophil/total neutrophil ratio > 0.2, white blood cell count < 5/nl, platelet count < 100/nl) and antibiotic treatment for ≥5 days, but no proof of causative agent in the blood culture. *Blood-culture proven sepsis* was defined as clinical sepsis with proof of causative agent in the blood culture. If *Coagulase negative staphylococci (CoNS)* were detected as single pathogen in the blood culture, a CrP value of > 1 mg/dl was mandatory to be defined as true *CoNS* sepsis. *Early onset sepsis* was defined as blood-culture confirmed sepsis occurring within the first 72 h after birth, *late onset sepsis* was defined as blood-culture confirmed sepsis after 72 h.

*Mortality* was defined as death during primary stay in hospital.

*Sepsis-related mortality* was defined as sepsis to be the most probable or confirmed cause of death as assigned by the attending neonatologist.

*Case fatality rate* was defined as percentage of infants dying from sepsis caused by the specific pathogen detected in the last blood culture (not in screening swabs) before death (numerator) from all infants suffering from sepsis caused by the same pathogen (denominator).

### Pathogens detected by KRINKO recommended colonization screening

According to the German commission for hospital hygiene and infection prevention (KRINKO) recommendations weekly colonization screening of infants with a birth weight < 1500 g in intensive care should be performed [11, 12]. This screening (throat and rectal swab) was performed according to local microbiological standards and should detect the following pathogens:

KRINKO I – all multi-drug resistant organisms (MDRO), i.e. Multidrug-resistant Gram negative bacteria (MRGN), 2MRGN (resistant to Piperacillin and Cefotaxim or Ceftazidim), 3MRGN (resistant to Piperacillin and Cefotaxim or Ceftazidim and fluorchinolones) and 4MRGN (resistant to Piperacillin and Cefotaxim or Ceftazidim and fluorchinolones and carbapenems); methicillin-resistant *Staphylococcus aureus (MRSA).*

KRINKO II – *Acinetobacter* spp., *Klebsiella* spp., *S.aureus* without antibiotic resistance patterns.

KRINKO III – pathogens with high epidemic potential but not MDRO including *Serratia marcescens*, *Pseudomonas aeruginosa, Klebsiella* and *Enterobacter* spp.

The following extended hygiene precautions are recommended based on screening results: barrier protection (gloves and gown) for all infants colonized with 2MRGN (KRINKO I), KRINKO II and III pathogens; isolation rooms for all infants with 3MRGN and 4MRGN and MRSA. No antibiotic treatment recommendations are given.

### Statistical analysis

Data analyses were performed using the SPSS 26.0 data analysis package (Munich, Germany). Study populations were compared using univariate analysis. Continuous variables were evaluated using t-test. Categorical variables were evaluated with Chi square and Fisher’s exact test. A *p*-value of < 0.05 was considered as statistically significant for single tests.

After univariate analyses, multivariate logistic regression models were used to identify independent risk factors of sepsis mortality. For multivariate logistic regression, only the last positive blood culture before discharge or death was taken into account. Odds ratios (OR) and 95% confidence intervals (CI) were calculated. A *p*-value of < 0.05 was considered statistically significant.

## Results

A total of 12.823 extremely preterm infants born in GNN centers were included in this study, 3.920 infants before publication of updated guidelines (treatment 2011–2013) and 8.903 infants after introduction of mandatory colonization screening (treatment 2014–2018). There were no differences in clinical characteristics in both groups (Table 1).

**Table 1:** Baseline characteristics of all extremely preterm infants born in GNN centres

Year of discharge	2011–2013 *n* = 3.920	2014–2018 *n* = 8.903	*p*
Gestational age [weeks, mean ± SD]	26.5 ± 1.6	26.5 ± 1.7	0.62*
Birth weight [grams, mean ± SD]	850 ± 253	852 ± 256	0.61*
Male gender (%, 95 CI)	53.8 (52.2–55.4)	53.5 (52.5–54.5)	0.58
Multiple birth (%, 95 CI)	29.1 (27.7–30.5)	31.9 (30.9–32.9)	0.002
Small for gestational age(%, 95 CI)	14 (13.0–15.1)	13.5 (12.8–14.2)	0.8

*p*-values were derived from * t-test or Fisher’s exact test

### Sepsis related mortality after introduction of colonization screening

Infants treated after guideline update had a lower mortality (12.5% vs. 13.8%, *p* = 0.036) and a reduced rate of clinical sepsis (31.4 vs. 42.8%, *p* <  0.001) and culture-proven sepsis (14.4% vs. 16.5%, *p* = 0.003; Table 2). Table 3 demonstrates a moderate decrease in culture-proven sepsis and clinical sepsis rates between 2011 and 2012. Other than that the differences in sepsis incidences and mortality occurred in timely association with guideline update. We noted a declining rate of Gram-positive sepsis (11.2% vs. 13.2%, *p* = 0.001) while the risk for Gram-negative sepsis (3.4%. vs. 3.8%, *p* = 0.26) was not decreased over time. Culture proven sepsis with pathogens detected by the weekly colonization screening was not different before versus after publication of the updated guideline, i.e. KRINKO I/MDRO (0.6% vs. 0.5%), KRINKO II (2.2 vs. 2.0%) and KRINKO III (1.1% vs. 1.1%).

**Table 2:** Sepsis and mortality before and after guideline update

Year of discharge	2011–2013 *n* = 3.920 (%, 95 CI)	2014–2018 *n* = 8.903 (%, 95 CI)	*p*#
Sepsis with positive blood culture	16.5 (15.3–17.6)	14.4 (13.7–15.1)	0.003
Clinical sepsis^a^	42.8 (41.1–44.6)	31.4 (30.4–32.4)	< 0.001
Early-onset sepsis^a^ with positive blood culture	1.7 (1.3–2.2)	1.4 (1.2–1.8)	0.3
Late-onset sepsis^a^ with positive blood culture	17.0 (15.7–18.4)	14.9 (14.0–15.8) %	0.008
Gram positive sepsis	13.2 (12.2–14.3)	11.2 (10.6–11.9) %	0.001
Gram negative sepsis	3.8 (3.2–4.4)	3.4 (3.0–3.8) %	0.26
KRINKO I/MDRO sepsis	0.5 (0.3–0.7)	0.6 (0.5–0.8) %	0.36
KRINKO II sepsis	2.0 (1.6–2.5)	2.2 (1.9–2.6) %	0.49
KRINKO III sepsis	1.1 (0.8–1.5)	1.1 (0.9–1.3) %	0.98
*Candida* sepsis	0.5 (0.3–0.7)	0.4 (0.3–0.6) %	0.52
Sepsis related mortality	2.3 (1.8–2.8)	2.1 (1.8–2.4) %	0.54
Total mortality (%, 95 CI)	13.8 (12.8–14.9)	12.5 (11.8–13.2) %	0.036

KRINKO I – all MDRO, KRINKO II – *Acinetobacter* spp., *Klebsiella* spp., *S. aureus* without MDRO; KRINKO III – pathogens with high epidemic potential but no MDRO i.e. *Serratia marcescens*, *Pseudomonas aeruginosa, Klebsiella* and *Enterobacter* spp. #Fisher’s exact test (two-sided)

^a^The data is based on the population of infants enrolled in the GNN (*n* = 2.948/6.630)

**Table 3:** Sepsis and mortality on an annual basis

Year of discharge	2011 *N* = 1.160%	2012 *N* = 1.409%	2013 *N* = 1.351%	2014 *N* = 1.632%	2015 *N* = 1.825%	2016 *N* = 1.710%	2017 *N* = 1.855%	2018 *N* = 1.881%
Sepsis with positive blood culture	17.8	15.4	16.4	14.6	14.9	13.8	14.8	13.8
Clinical sepsis^a^	47.5	41.3	40.7	37.6	38.2	28.9	28.5	27.0
Early-onset sepsis^a^ with positive blood culture	1.7	1.4	2.1	1.5	1.7	1.1	1.7	1.2
Late-onset sepsis with positive blood culture^a^	18.6	16.5	16.3	15.5	15.8	13.9	15.3	13.8
Gram positive sepsis	14.2	12.4	13.2	11.0	12.1	10.3	11.8	11.0
Gram negative sepsis	4.6	3.5	3.4	3.8	3.2	3.7	3.2	3.2
KRINKO I/MDRO sepsis	0.3	0.5	0.6	1.0	0.4	0.6	0.4	0.7
KRINKO II sepsis	2.4	1.9	1.9	2.9	2.3	2.2	2.0	1.9
KRINKO III sepsis	1.4	1.2	0.7	1.0	1.2	1.3	1.0	1.0
*Candida* sepsis	0.3	0.6	0.5	0.8	0.4	0.6	0.2	0.2
Sepsis related mortality	2.8	1.9	2.2	2.6	1.9	2.3	1.8	2.0
Total mortality	16.0	12.8	13.0	12.6	12.1	12.7	12.0	13.1

^a^For clinical sepsis, and early/late onset sepsis the data is based on the population of infants enrolled in the GNN

Significant changes in the detection rate of bacterial pathogens were noted only for pathogens that are not targeted by screening; i.e. non MDRO *E.coli* (after vs. before guideline update: 1.2 vs. 1.8%, *p* = 0.017), *Staphylococcus epidermidis* (5.7 vs. 8.1%, *p* <  0.001) and *Group B Streptococcus* (0.6 vs. 0.9%, *p* = 0.035; Suppl. Table 1). While the incidence for late-onset sepsis declined over time, early or late-onset sepsis related mortality were not significantly influenced by guideline update (Suppl. Table II).

### Exposure to antibiotics

The exposure to antibiotics, in particular the use of penicillins and glycopeptide antibiotics was reduced between 2014 and 2018 and 2011–2013 (Table 4). Notably, the exposure to infants with cefotaxime declined after guideline update (31 vs. 41%, *p* <  0.001), while infants were more frequently exposed to meropenem (31.6 vs. 26.3%, *p* <  0.001). The exposure rate to any carbapenem was not significantly different (35.4 vs. 33.4%, *p* = 0.06). We also noted temporal changes for the use of antifungals, i.e. an increased exposure to fluconazole and liposomal amphothericin B after guideline update (Table 4).

**Table 4:** Exposure to anti-infective drugs before and after guideline update

Year of discharge	2011–2013 *n* = 2.948%	2014–2018 *n* = 6.630%	*P*#
Any antibiotics	96.7	93.3	< 0.001
Penicillins	76.6	66.6	< 0.001
Ampicillin	71.0	66.3	< 0.001
Piperacillin	15.6	17.5	0.02
Piperacillin/Tazobactam	11.5	12.0	0.65
Aminoglycosides	75.3	74.1	0.2
Gentamycin	56.0	56.9	0.44
Tobramycin	20.6	18.3	0.01
Glycopeptides	53.4	49.2	< 0.001
Vancomycin	42.0	41.0	0.34
Teicoplanin	11.8	9.5	0.001
Carbapenems	33.4	35.4	0.06
Meropenem	26.3	31.6	< 0.001
Imipenem	9.1	4.9	< 0.001
Others			
Cefotaxim	40.1	31.1	< 0.001
Metronidazol	6.4	5.4	0.05
Erythromycin	11.8	9.3	0.001
Fluconazol	4.4	9.5	< 0.001
Amphothericin B	1.7	3.5	< 0.001

*Exposure to anti-infective drugs* was defined as treatment of preterm infants (number of neonates who got any dose of the according anti-infective drug; denomination: number of infants admitted and enrolled in GNN) for clinical suspicion of infection during the initial stay in hospital

# Fisher’s exact test (two-sided). All Table 4 data is based on infants enrolled in the GNN

### Pathogen-specific case fatality rate

Nine pathogens were significantly associated with sepsis related death in univariate analyses. *Pseudomonas aeruginosa* had the highest case-fatality rate [8/16, 50%, OR 47 (17–126), *p* <  0.001)] followed by *Candida albicans* [13/55, 24%, OR 15 (7.8–28), *p* <  0.001], *E. coli* [19/181, 11%, OR 5.6 (3.5–9.2), *p* <  0.001)], extended spectrum ß-lactamase (ESBL) *E. coli* [4/37, 11%, OR 5.6 (2.0–16), *p* = 0.01], *Klebsiella* spp. [9/83, 11%, OR 5.4 (2.6–11.2), *p* <  0.0001], *Group B Streptococcus* [8/88, 9.1%, OR 4.7 (2.2–9.7), *p* = 0.001], *Enterococci* spp. [9/114, 7.9%, OR 4.0 (2.0–8.0), *p* = 0.001], *Enterobacter* spp. [6/108, 5.6%, OR 2.7 (1.2–6.2), *p* <  0.0001] and *Staphylococcus haemolyticus* [12/220, 5.5%, OR 2.7 (1.5–4.8), *p* = 0.003]. In multivariate logistic regression models for sepsis related mortality we included “treatment after guideline update” as intervention and known confounding factors such as gestational age, small for gestational age, gender and multiple birth. For the regression analysis of pathogen-specific case fatality, we only included the pathogen found in the last positive blood culture before discharge or death in order to exclude the possibility that sepsis related mortality is attributed to specimens of previous blood-cultures. Nine pathogens of culture-proven sepsis were associated with sepsis-related death, e.g. *Pseudomonas aeruginosa* sepsis had the highest risk for sepsis related death [OR 59 (19–180), *p* <  0.001)]. However, the guideline update was neither associated with sepsis-related mortality in univariate analysis (OR 0.92, 95% CI 0.72–1.19) nor with pathogen-specific case fatality (Table 5).

**Table 5:** Mortality due to sepsis and blood culture results

Exposure	N exposed	Mortality due to sepsis [%]	Odds ratio* (95% CI)	p*
Gestational age [OR per additional week]	12.823	2.2	0.7 (0.66–0.75)	< 0.001
Discharge 2014–2018	8.903	2.1	0.9 (0.73–1.23)	0.69
Female sex	5.950	1.8	0.7 (0.58–0.95)	0.02
Multiple birth	3.979	2.0	0.9 (0.70–1.22)	0.59
SGA	1.753	4.1	2.0 (1.49–2.58)	< 0.001
All blood cultures negative*	10.893	1.5	Ref.	
*Staph. epidermidis*	745	2.4	1.2 (0.7–2.0)	0.74
*Staph. haemolyticus*	196	5.1	2.6 (1.3–5.0)	0.005
*E coli*	169	11.2	7.3 (4.3–12)	< 0.001
*Staph. aureus*	150	4.0	2.3 (1.00–5.3)	0.053
*Staph. capitis*	105	0	–	
*Enterococci, no VRE*	97	8.2	4.2 (2.0–9.0)	< 0.001
*Enterobacter spp.*	94	6.4	3.4 (1.4–7.9)	0.005
*Group B Strep.*	86	9.3	5.5 (2.6–12)	< 0.001
*Klebsiella, no MDRO*	75	10.7	5.6 (2.6–12)	< 0.001
*Candida spp*	45	26.7	16 (7.9–32)	< 0.001
*E. coli ESBL*	32	12.5	6.7 (2.3–20)	0.001
*Staph. hominis*	28	7.1	4.0 (0.9–17)	0.06
*Enterococci, VRE*	20	5.0	2.8 (0.4–21)	0.33
*Serratia*	17	5.9	2.9 (0.4–23)	0.30
*Klebsiella ESBL*	16	0	–	
*MRSA*	16	6.3	3.7 (0.5–28)	0.21
*Pseudomonas*	14	57.1	59 (19–180)	< 0.001
*Other streptococci*	12	0	–	
*Listeria*	8	12.5	11 (1.3–92)	0.04
*Pneumococci*	3	0	–	
*Proteus*	1	0	–	
*Bacteroides*	1	0	–	

^*^Logistic regression, result of last positive blood culture before death or discharge was entered as category variable with all blood-culture negative infants as reference

## Discussion

The GNN collaboration offers a great opportunity to compile data about interventions taken and to evaluate clinical practice. We found a temporal decline of clinical sepsis, total culture-proven sepsis and late-onset sepsis incidence. However, introduction of weekly colonization screening had no effects on sepsis-related mortality and sepsis with MDRO or highly epidemic pathogens. While the exposure rate to cefotaxime declined, the use of meropenem increased. Recent survey data indicate that the compliance to KRINKO recommendations is high among German NICUs (> 90% for MDRO screening) [21, 22], hence our cohort is a representative sample of the population of extremely preterm infants in Germany.

The advances in neonatology and participation in our network led to declining mortality during the observational period. In comparison to other network studies, the survival rate of infants is relatively high, i.e. 75% in infants born at 24 weeks of gestation vs. 84% (Japan), 71% (Finland), 70% (Sweden) and 58% (USA) [2, 23, 24]. After respiratory failure, sepsis remains the second most important cause of death, e.g. 17% deaths in a recent GNN sample in infants < 29 weeks [2]. Notably, the incidence of clinical or culture-proven (late-onset) sepsis has been continuously decreasing which is mainly explained by a decline in the frequency of infections with Coagulase negative staphylococci but not with a reduced number of Gram-negative infections. In that aspect, weekly colonization screening has no specific impact but might be considered as part of an increased “sepsis awareness” bundle in NICUs which has surrogate positive effects on sepsis-related outcome. Whether improved sepsis outcome is related to extended hygiene precautions and whether the screening strategy is a cost-effective measure remains a matter of discussion.

No difference was observed for pathogen-specific fatality rates before and after guideline update. The sepsis pathogens have different habitats and pathogenicity factors which lead to variable clinical courses. In this study, every second infant with *Pseudomonas aeruginosa* infection died which is in line with previous reports [25, 26]. The case-fatality rates for *Candida albicans* (24%), *E. coli* (11%), *Klebsiella* spp. (11%) and *Group B Streptococcus* (9%) were also significant. In a recent Italian area-based, retrospective study culture-proven late-onset sepsis occurred in 12% of preterm ELBWI and case fatality rate was 13% [27] which is also comparable to Swiss data (12%) [7].

Survivors of sepsis have a major risk for long-term morbidity of several organ systems including CNS, lung and gut [1]. Given the lack of reliable biomarkers to discriminate between infection and other causes of systemic inflammation, empirical broad-spectrum antibiotics are administered when sepsis is clinically suspected. The choice of empiric first line therapy is of critical importance in a patient colonized with an MDRO, as a therapeutic mismatch during the first 2 to 3 days of the infection (until culture results are reported) may be the most important reason for a complicated course of sepsis. On the other hand, the uncritical use of carbapenems which has been noted in timely association with public media reports on NICU infection outbreaks [10] should be avoided. In our setting, the active continuous surveillance did not influence Gram-negative/MDRO sepsis related outcomes but led to an increased prescription rate of meropenem. Hence there is a perceived threat posed by MDRO which is not supported by epidemiological data. The latter aspect and the increasing exposure of extremely preterm infants to antifungal drugs need to become major targets for anti-infective stewardship programs which have recently been implemented in several GNN NICUs. The effectiveness of network feedback and benchmarking on drug prescriptions has been shown for the use of cefotaxime. The prescription rate of cefotaxime declined during the observational period in this study, after continued education on the risks of cefotaxime, e.g. of selection of MDR enterobacteria has been provided during GNN meetings. In line with that, we noted a 3.4% reduction in the total antibiotic treatment rate. i.e. after guideline update 225 more extremely preterm infants were not exposed to antibiotics at all.

Interpretation of surveillance data is an interdisciplinary task involving experts in neonatology, infectious diseases, microbiology, hospital hygiene and pharmacy. Continuous surveillance allows a characterization of the local epidemiology in longer time periods, the investigation of the epidemic potential of specific pathogens and an evaluation of outbreaks including a root-cause analysis. We provide the first robust data that the availability of screening data is associated with declining sepsis rates probably due to an increased risk awareness. Routine screening followed by extended barrier precautions for colonized babies is time consuming and does not necessarily have an effect on the frequency of invasive disease or mortality from sepsis with screening relevant pathogens. This needs to be put in into the perspective of an increased use of third-line anti-infective drugs, staff intensity, costs and decisions to the disadvantage of other priorities of care (e.g. avoidance of co-bedding of twins with different screening results). It has also been debated whether the care of MDRO colonized babies in isolation rooms with closed doors without permanent presence of a nurse might actually increase the risk, e.g. to suffer from prolonged apnoea, specifically in times of overcrowding and understaffing.

Our study has limitations. The observational design does not allow to account for all confounding factors that might have an impact on change over time (i.e. number of infants enrolled/year, center-specific effects, different local guidelines before mandatory implementation of guidelines). In addition, although none of the NICUs had introduced a generalized screening that was postulated by the KRINKO, some NICUs used selective screening approaches at least in outbreak situations which might have influenced the results reported in ours study. Second, we were not able to determine the compliance rate to extended hygiene precautions based on screening results in our study. Finally, the treatment rate with antibiotics is a rather crude estimation of the actual exposure, i.e. the documentation of defined daily doses of antibiotics is needed for future evaluations.

## Conclusion

In conclusion, the introduction of weekly screening and extended hygiene measures is associated with reduced sepsis rates, but has no effects on sepsis-related mortality and sepsis with screening-relevant pathogens. Every effort should be made to prevent colonization and nosocomial transmission with potentially life-threatening bacteria. Colonized infants represent the most important reservoir for subsequent nosocomial transmission. Continuous surveillance is regarded as an important tool for hospital hygiene management but has not yet been proven to be effective to reduce sepsis-related outcome in highly preterm infants. Additional assessments (genotyping) need to be performed to evaluate the identity of colonizing and invasive strains in outbreak situations. Unwanted effects of screening need to be actively addressed by antibiotic stewardship programs with impactful interventions [28]. Future studies should focus on protective measures like modulation of the local microbiome promoting gut eubiosis in order to control drug-resistant or highly epidemic enterobacteria.

## Supplementary information

**Additional file 1: Supplementary Table I.** Bacterial sepsis pathogens before and after guideline update

**Additional file 2: Supplementary Table II.** Early onset sepsis, late onset sepsis and mortality

## Data Availability

The datasets used and/or analysed during the current study are available from the corresponding author on reasonable request.

## Abbreviations

EXTREMELY PRETERM INFANTS: Extremely low gestational age newborns
ESBL: Extended spectrum beta-lactamase
GA: Gestational age
GBS: Group B streptococci
GNN: German Neonatal Network
LOS: Late onset sepsis
MDRO: Multi drug resistant organisms
MRSA: Methicillin resistant *Staphylococcus aureus*
NEC: Necrotizing enterocolitis
NICU: Neonatal intensive care unit
VRE: Vancomycin resistant enterococci

## References

[1] Stoll BJ, Hansen NI, Bell EF, Walsh MC, Carlo WA (2015). Trends in care practices, morbidity, and mortality of extremely preterm neonates, 1993-2012. JAMA..

[2] Humberg A, Härtel C, Rausch TK, Stichtenoth G, Jung P (2020). German neonatal network. Active perinatal care of preterm infants in the German Neonatal Network. Arch Dis Child Fetal Neonatal Ed.

[3] Tarr PI, Warner BB (2016). Gut bacteria and late-onset neonatal bloodstream infections in preterm infants. Semin Fetal Neonatal Med.

[4] Shane AL, Sanchez PJ, Stoll BJ (2017). Neonatal sepsis. Lancet..

[5] Gkentzi D, Kortsalioudaki C, Cailes BC, Zaoutis T, Kopsidas J (2019). Epidemiology of infections and antimicrobial use in Greek neonatal units. Arch Dis Child Fetal Neonatal Ed.

[6] Cailes B, Kortsalioudaki C, Buttery J, Pattnayak S, Greenough A (2018). neonIN network. Epidemiology of UK neonatal infections: the neonIN infection surveillance network. Arch Dis Child Fetal Neonatal Ed.

[7] Giannoni E, Agyeman PKA, Stocker M, Posfay-Barbe KM, Heininger U (2018). Swiss pediatric sepsis study. Neonatal sepsis of early onset, and hospital-acquired and community-acquired late onset: A prospective population-based cohort study. J Pediatr.

[8] Härtel C, Faust K, Avenarius S, Bohnhorst B, Emeis M (2012). German neonatal network (GNN). Epidemic microclusters of blood-culture proven sepsis in very-low-birth weight infants: experience of the German Neonatal Network. PLoS One.

[9] Reichert F, Piening B, Geffers C, Gastmeier P, Bührer C, Schwab F (2016). Pathogen-specific clustering of nosocomial blood stream infections in very preterm infants. Pediatrics..

[10] Härtel C, Hartz A, Bahr L, Gille C, Gortner L (2016). German Neonatal Network Media stories on NICU outbreaks lead to an increased prescription rate of third-line antibiotics in the community of neonatal care. Infect Control Hosp Epidemiol.

[11] Kommission für Krankenhaushygiene und Infektionsprävention beim Robert Koch-Institut (2013). Praktische Umsetzung sowie krankenhaushygienische und infektionspräventive Konsequenzen des mikrobiellen Kolonisationsscreenings bei intensivmedizinisch behandelten Früh- und Neugeborenen – Ergänzende Empfehlung der KRINKO beim Robert Koch-Institut, Berlin, zur Implementierung der Empfehlungen zur Prävention nosokomialer Infektionen bei neonatologischen Intensivpflegepatienten mit einem Geburtsgewicht unter 1500g aus dem Jahr 2007 und 2012 [GERMAN]. German commission for hospital hygiene and infection prevention at Robert-Koch-Institute. Implementation of hospital hygiene and preventive consequences of the colonization screening for preterm and term infants in neonatal intensive care – additional recommendation for the implementation of recommendations for prevention of infections in very-low-birth-weight infants, from 2007 and 2012. Epidemiol Bull.

[12] Dame C, Christoph J, Eckmanns T, Gärtner B, Geffers C, et al. Risikocharakterisierung intensivmedizinisch behandelter Früh- und Neugeborener und Daten zur Ist-Situation in deutschen neonatologischen Intensivpflegestationen 2013 - Fachliche Erläuterungen zu folgender Empfehlung: Praktische Umsetzung sowie krankenhaushygienische und infektionspräventive Konsequenzen des mikrobiellen Kolonisationsscreenings bei intensivmedizinisch behandelten Früh- und Neugeborenen Ergänzende Empfehlung der Kommission für Krankenhaushygiene und Infektionsprävention (KRINKO) beim Robert Koch-Institut, Berlin zur Implementierung der Empfehlungen zur Prävention nosokomialer Infektionen bei neonatologischen Intensivpflegepatienten mit einem Geburtsgewicht unter 1.500 g aus dem Jahr 2007 und 2012. [GERMAN]. Risk characterization of preterm and term infants in newborn intensive care and benchmark data in German NICUs 2013. Explanations for the implementation of hospital hygiene and preventive consequences of the colonization screening for preterm and term infants in neonatal intensive care – additional recommendation for the implementation of recommendations for prevention of infections in very-low-birth-weight infants, from 2007 and 2012. Epidemiol Bull. 2013;42(Suppl):1–12.

[13] Folgori L, Tersigni C, Hsia Y, Kortsalioudaki C, Heath P (2018). The relationship between gram-negative colonization and bloodstream infections in neonates: a systematic review and meta-analysis. Clin Microbiol Infect.

[14] Stapleton PJM, Murphy M, McCallion N, Brennan M, Cunney R, Drew RJ (2016). Outbreaks of extended spectrum beta-lactamase-producing Enterobacteriaceae in neonatal intensive care units: a systematic review. Arch Dis Child Fetal Neonatal Ed.

[15] Seidel J, Haller S, Eckmanns T, Harder T (2018). Routine screening for colonization by Gram-negative bacteria in neonates at intensive care units for the prediction of sepsis: systematic review and meta-analysis. J Hosp Infect.

[16] Benenson S, Levin PD, Block C, Adler A, Ergaz Z (2012). Continuous surveillance to reduce extended-spectrum β-lactamase Klebsiella pneumoniae colonization in the neonatal intensive care unit. Neonatology..

[17] Wisgrill L, Zizka J, Unterasinger L, Rittenschober-Böhm J, Waldhör T (2017). Active surveillance cultures and targeted decolonization are associated with reduced methicillin-susceptible *Staphylococcus aureus* infections in VLBW infants. Neonatology.

[18] Francis S, Khan H, Kennea N (2012). Infection control in United Kingdom neonatal units: variance in practice and the need for an evidence base. J Infect Prev.

[19] Anthony M, Bedford-Russell A, Cooper T, Fry C, Heath PT (2013). Managing and preventing outbreaks of gram-negative infections in UK neonatal units. Arch Dis Child Fetal Neonatal Ed.

[20] Leistner R, Piening B, Gastmeier P, Geffers C, Schwab F (2013). Nosocomial infections in very low birthweight infants in Germany: current data from the National Surveillance System NEO-KISS. Klin Padiatr.

[21] Litz JE, Goedicke-Fritz S, Härtel C, Zemlin M, Simon A (2019). Management of early- and late-onset sepsis: Results from a survey in 80 German NICUs. Infection..

[22] Litz JE, Goedicke-Fritz S, Härtel C, Wagenpfeil G, Zemlin M, Simon A, Litz JE, Goedicke-Fritz S, Härtel C, Wagenpfeil G, Zemlin M, Simon A (2019). Umsetzung des mikrobiologischen Kolonisationsscreenings: Umfrage an 80 neonatologischen Intensivstationen. Epid Bull.

[23] Helenius K, Sjörs G, Shah PS, Modi N, Reichmann B (2017). International Network for Evaluating Outcomes (iNeo) of Neonates. Survival in very preterm infants: an international comparison of 10 national neonatal networks. Pediatrics.

[24] Younge N, Goldstein RF, Bann CM, Hintz SR, Patel RM (2017). Eunice Kennedy Shriver National Institute of Child Health and Human Development neonatal research network. Survival and neurodevelopmental outcomes among periviable infants. N Engl J Med.

[25] Leigh L, Stoll BJ, Rahman M, McGowan J (1995). Pseudomonas aeruginosa infection in very low birth weight infants: a case-control study. Pediatr Infect Dis J.

[26] Greenberg RG, Kandefer S, Do BT, Smith PB, Stoll BJ (2017). Eunice Kennedy Shriver National Institute of Child Health and Human Development Neonatal Research Network. Late-onset sepsis in extremely premature infants: 2000-2011. Pediatr Infect Dis J.

[27] Berardi A, Sforza F, Baroni L, Spada C, Ambretti S (2019). Epidemiology and complications of late-onset sepsis: an Italian area-based study. PLoS One.

[28] Ran NC, van den Hoogen A, Hemels MAC (2019). Gram-negative late-onset sepsis in extremely low birth weight infants is emerging in the Netherlands despite quality improvement programs and antibiotic stewardship!. Pediatr Infect Dis J.

