# Supplementary material for: Sepsis related mortality of extremely low gestational age newborns after the introduction of colonization screening for multi-drug resistant organisms
Source: Antimicrob Resist Infect Control. 2020 Aug 26;9:144. doi: 10.1186/s13756-020-00804-8 (PMC7449086; doi:10.1186/s13756-020-00804-8)
Supplement: Supplementary file 1 — Additional file 1: Supplementary Table I. Bacterial sepsis pathogens before and after guideline update [file 13756_2020_804_MOESM1_ESM.docx]

**Supplementary table I:** Bacterial sepsis pathogens before and after guideline update

| Year of discharge | 2011 – 2013 n = 3.920  % | 2014 – 2018 n = 8.903  % | p* |
| --- | --- | --- | --- |
| Gram positive bacteria |  |  |  |
| *Staph. epidermidis* | 8.1 | 5.7 | < 0.001 |
| *Staph. haemolyticus* | 1.6 | 1.8 | 0.53 |
| *Staph. aureus* | 1.3 | 1.5 | 0.35 |
| *MRSA* | 0.2 | 0.1 | 0.44 |
| *Staph. capitis* | 1.0 | 0.9 | 0.36 |
| *Staph. hominis* | 0.2 | 0.3 | 0.6 |
| *Enterococci, no VRE* | 0.9 | 0.9 | 0.86 |
| *Enterococci, VRE* | 0.1 | 0.2 | 0.21 |
| *Group B Strep.* | 0.9 | 0.6 | 0.035 |
| *Other streptococci* | 0.04 | 0.1 | 0.25 |
| *Listeria* | 0 | 0.8 | - |
| *Pneumococci* | 0.02 | 0.02 | 1.0 |
| Gram negative bacteria |  |  |  |
| *E coli, no MDRO* | 1.8 | 1.2 | 0.017 |
| *E coli, MDRO* | 0.2 | 0.3 | 0.22 |
| *Enterobacter* | 0.8 | 0.9 | 0.67 |
| *Klebsiella, no MDRO* | 0.7 | 0.6 | 0.48 |
| *Klebsiella, MDRO* | 0.1 | 0.2 | 0.37 |
| *Serratia spp.* | 0.2 | 0.1 | 0.8 |
| *Pseudomonas aeruginosa* | 0.2 | 0.1 | 0.55 |

* Fisher’s exact test (two-sided)
