# Supplementary material for: Sepsis related mortality of extremely low gestational age newborns after the introduction of colonization screening for multi-drug resistant organisms
Source: Antimicrob Resist Infect Control. 2020 Aug 26;9:144. doi: 10.1186/s13756-020-00804-8 (PMC7449086; doi:10.1186/s13756-020-00804-8)
Supplement: Supplementary file 2 — Additional file 2: Supplementary Table II. Early onset sepsis, late onset sepsis and mortality [file 13756_2020_804_MOESM2_ESM.docx]

**Supplementary table II:** Early onset sepsis, late onset sepsis and mortality

| Year of discharge | 2011 – 2013  n = 2.948  (n / %) | 2014 – 2018  n = 6.630  (n / %) | p* |
| --- | --- | --- | --- |
| Early onset sepsis | 51 / 1.7 | 96 / 1.4 | 0.32 |
| Total mortality after early onset sepsis | 9/51 17.6 | 16/96 16.7 | 1.00 |
| Mortality due to sepsis after early onset sepsis | 4/51 7.8 | 8/96 8.3 | 1.00 |
| Late onset sepsis | 502 / 17 | 982 / 14.8 | 0.006 |
| Total mortality after late onset sepsis | 39/502 7.8 | 85/982 8.7 | 0.62 |
| Mortality due to sepsis after late onset sepsis | 12/502 2.4 | 29/982 3.0 | 0.62 |

The data is based on infants enrolled in the GNN, * Fisher´s exact test (two-sided)
